# Extensive karyotype variability of African fish genus *Nothobranchius* (Cyprinodontiformes)

**DOI:** 10.3897/CompCytogen.v12i3.25092

**Published:** 2018-09-10

**Authors:** Eugene Krysanov, Tatiana Demidova

**Affiliations:** 1 Severtsov Institute of Ecology and Evolution, Russian Academy of Sciences, Moscow, 119071 Russia Severtsov Institute of Ecology and Evolution, Russian Academy of Sciences Moscow Russia

**Keywords:** African killifishes, fish cytogenetics, karyotype differentiation

## Abstract

Karyotypes of 65 species of the genus *Nothobranchius* Peters, 1868 were reviewed and of those 35 examined first time. The results of present study have shown that fishes of the genus *Nothobranchius* possessed highly diverse karyotypes. The diploid chromosome number (2n) ranged from 16 to 50. The most frequent 2n was 2n = 36 (in 35 species) while the second one 2n = 38 (in 13 species). Proportion of biarmed chromosomes varied from 0 to 95% between species. Diploid chromosome number variability apparently exists as a result of chromosomal fusions or fissions and extensive karyotypic formula alterations promoting by inversions. Multiple sex chromosomes of system X_1_X_1_X_2_X_2_/X_1_X_2_Y type were found only in karyotypes of 5 species. The extensive karyotype variability, unusual for teleosts, of genus *Nothobranchius* can be likely associated with the characteristics of its life cycle and inhabiting under unstable environment of East African savannah temporal pools.

## Introduction

More than a half of teleost fish examined had diploid chromosomes number 2n = 48-50 (Mank and Avise 2006, Molina et al. 2014). Karyotypes containing either high or low proportions of acrocentrics tend to be more frequent than those with balanced numbers of acrocentric and metacentric chromosomes (Molina et al. 2014). According to Naruse et al. 2004, Galetti et al. 2006 and Molina et al. 2014 the karyotype of teleost fishes is stable but intrachromosomal rearrangements such as inversions and centromere shift are common. The association of chromosome rearrangements with speciation is known, especially inversions which can promote the local adaptation due to suppression of recombination and thus accumulation of linked adaptive genes. These then favour the accumulation of genetic incompatibilities between species, reduce fertility of hybrids contributing to reproductive isolation and speciation (Navarro and Barton 2003, Kirkpatrick and Barton 2006, Noor et al. 2001, Rieseberg 2001, Hooper & Price 2017). A higher degree of karyotype variation for freshwater fish species inhabiting a more unstable environment compared to that of marine ones has been demonstrated (Nirchio et al. 2014).

Killishes of the genus *Nothobranchius* Peters, 1868 comprise 76 valid species (Eschmeyer et al. 2018, FishBase 2018). The main life-style characteristics of killifishes reside in fact that species and their populations inhabiting in ephemeral pools of East Africa are isolated both geographically and temporarily due to extremely short life cycle (Wildekamp 2004; Reichard 2016).

Phylogenetic data based on molecular markers demonstrated that the genus *Nothobranchius* is a monophyletic assemblage and it includes four geographically separated clades (Dorn et al. 2014). Costa (2018) performed taxonomy analysis of the genus on the basis of morphology and phylogenetic data. Six subgenera were recognised: *Adiniops* Myers, 1924, *Cynobranchius* Costa, 2018, *Nothobranchius* Peters, 1868, *Paranothobranchius* Seegers, 1985, *Plesiobranchius* Costa, 2018, and *Zononothobranchius* Radda, 1969.

Karyotypes of 30 species were described earlier and high karyotype variability was revealed (summarized in Arai 2011). The diploid chromosome number (2n) of *Nothobranchius* species varies from 16 to 50 (Scheel 1990, Krysanov et al. 2016). Two species *N.guentheri* (Pfeffer, 1893) and *N.brieni* Poll, 1938 had multiple chromosome system (Ewulonu et al. 1985, Krysanov et al. 2016). Thus, the representatives of the genus *Nothobranchius* is a good model for studying karyotype differentiation due to high karyotype variability and features of the life cycle.

The aim of the study was to characterize karyotype diversity of the genus *Nothobranchius* and conduct cytogenetic comparison among different species. In present study, we i) reviewed all available data dealing with cytogenetic study of *Nothobranchius* species and ii) analyzed 35 other species not studied as yet for 2n and karyotype composition using conventional cytogenetic protocol.

## Material and methods

### Specimens collection

Individuals of *Nothobranchius* species were collected either from wild populations of East Africa or provided by killifish hobbyists. Geographical data and coordinates are given in supplements.

### Cytogenetic analysis

Chromosomes were prepared according to the method of Kligerman and Bloom (1977). The chromosome preparations were obtained from anterior kidney tissue. Briefly, individuals were injected intraperitoneally with 0.1% colchicine solution for 3-4 hours. The hypotonization in 0.075 M KCl was 20-30 min at room temperature. Then tissue samples were fixed in 3:1 methanol: acetic acid for 24 hours.

Slides were air dried and then stained with 2% Giemsa solution in phosphate buffer a (pH 6.8) for 10 min. Chromosomes were analyzed under microscope “AxioImager” Karl Zeiss (Germany) equipped with CCD camera and “KaryoImage” Metasystems Software (Germany). Chromosome morphology was determined according to Levan et al. (1964) and classified as metacentric (m), submetacentric (sm), subtelocentric (st) and acrocentric (a). To determine the fundamental number (NF), chromosomes of the m and sm groups were considered biarmed and those of group st/a uniarmed.

Statistical analysis was done using IBM SPSS 20 package. Data were tested for normality. Regression between the rate of biarmed chromosomes and diploid chromosome number, and the Spearman correlation were calculated.

## Results

**Figure 1.**
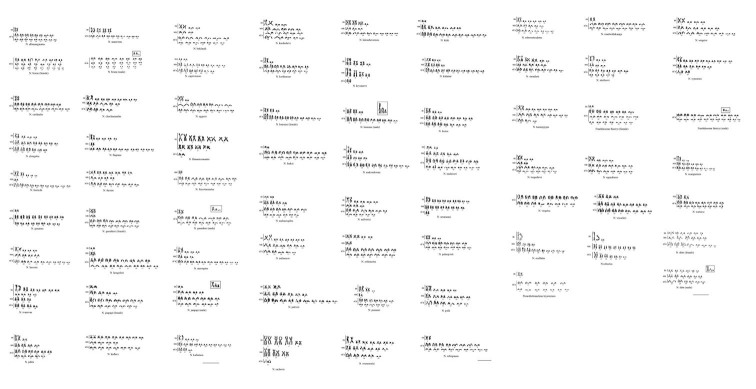
Karyotypes of species *Nothobranchius*. Scale bar: 10 μ.

Karyological data of 65 species of the genus *Nothobranchius* and two species of sister taxa *Fundulosoma* Ahl, 1924 and *Pronothobranchius* Radda, 1969 (according to Costa, 2018) are provided in Table 1 and Fig. 1.

**Figure 2.**
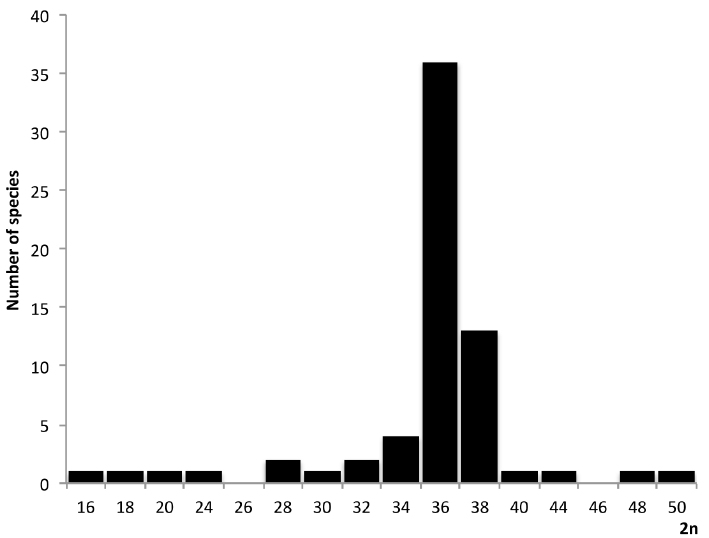
Histogram of the distribution of the diploid chromosome number (2n) in the genus *Nothobranchius*.

As evident, the number and morphology of chromosomes varied widely between karyotypes of analyzed species 2n ranged from 16 to 50 where the most frequent was 2n = 36 and second 2n = 38 (Fig. 2).

**Figure 3.**
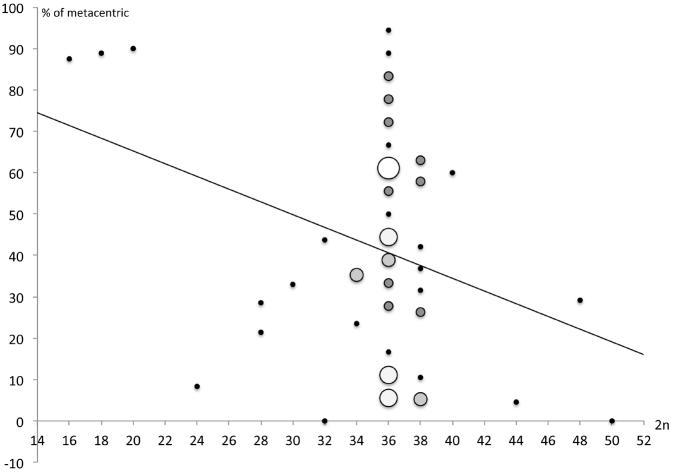
Scatter-plot of a diploid chromosome number (2n) and proportion of metacentric chromosomes with overall regression line. The diameter and color of circle indicate number of species from 1 to 5.

Our data showed that the proportion of biarmed chromosomes in the karyotype of the species varied widely from 0 to 95%. Regression between the rate of biarmed chromosomes and 2n was y = -1.607x + 96.863, R^2^ = 0.29 and the Spearman correlation was Rs = -0.181 (Fig. 3).

### Subgenus Cynobranchius

Karyotypes of two species belonging to this subgenus were described by Scheel (1981, 1990). The karyotype of *N.microlepis* had the 2n = 24 and most chromosomes in the karyotype were uniarmed with only one pair of biarmed chromosomes (NF = 26). *N.fasciatus* had 2n = 34 with 22 uniarmed and 12 biarmed chromosomes.

### Subgenus *Plesiobranchius*

The only species in the subgenus *N.virgatus* has 2n = 32 uniarmed chromosomes (NF = 32).

### Subgenus Nothobranchius

Four species *N.furzeri*, *N.kadleci*, *N.orthonotus* and *N.kuhntae* possesed the 2n = 38. Biarmed elements dominated in karyotypes of *N.kadleci* (NF = 62) and *N.furzeri* (NF = 60), and uniarmed chromosomes dominated in karyotypes of *N.kuhntae* (NF = 52) and *N.orthonotus* (NF = 48).

The karyotype of *N.pienaari* had 2n = 24 and most of chromosomes were uniarmed (NF = 42).

The lowest 2n was found in two closely related species *N.rachovii* (2n = 16, NF = 30) and *N.krysanovi* (2n = 18, NF = 34). Most of chromosomes in their karyotypes were metacentric elements with only one pair of acrocentric chromosomes as described earlier (Scheel 1990, Shidlovskiy at al. 2010). Both species had similar karyotype structure and were distinguished by one additional pair of metacentric chromosomes in *N.krysanovi*.

### Subgenus Paranothobranchius

The only species in the subgenus *N.ocellatus* has 2n = 30 and uniarmed chromosomes dominated in the karyotype (NF = 40).

### Subgenus Zononothobranchius

There are species in the subgenus possessing 2n higher than 38. The highest 2n = 49/50 among studied species was discovered in *N.brieni* (Krysanov et al. 2016) where all autosomes in the karyotype were acrocentric (NF = 50). *N.brieni* had karyotype with differentiated heteromorphic sex chromosomes X_1_X_1_X_2_X_2_/X_1_X_2_Y type (Krysanov et al. 2016). The karyotype of *N.malaissei* had diploid numbers 2n = 48 and uniarmed chromosomes dominated in the karyotype (NF = 62).

*N.milvertzi* had the 2n = 38 with karyotype formulae 10m+6sm+22st/a (NF = 54).

The rest species in subgenus had diploid chromosome numbers 2n = 36 (see table 1). The ratio of uniarmed and biarmed chromosomes differed among species. The most uniarmed chromosomes number was found for *N.boklundi* (NF = 46) which had 26 uniarmed and 10 biarmed chromosomes (6m+4sm+26st/a) and the least uniarmed chromosomes number was found for *N.neumanni* (NF = 70) with only two uniarmed and 34 biarmed chromosomes (18m+16sm+2st/a). Other species had karyotypes with uniformly decreasing numbers of uniarmed chromosomes from 24 to 4 and numbers of biarmed chromosomes increased correspondingly.

### Subgenus Adiniops

Eight species had the 2n = 38 with different ratio of uniarmed and biarmed chromosomes. Karyotypes of three species *N.fuscotaeniatus, N.geminus* and *N.luekei* possessed 36 uniarmed and only two biarmed chromosomes (NF = 40) while *N.vosseleri* (NF = 60) karyotype had only 16 uniarmed and 22 biarmed chromosomes. Other species had karyotypes with uniformly decreasing numbers of uniarmed chromosomes from 34 to 26 and numbers of biarmed chromosomes increased correspondingly. Females of *N.janpapi* had more chromosome than males 2n = 38/37 and multiple sex chromosome system X_1_X_1_X_2_X_2_/X_1_X_2_Y type was revealed.

The modal diploid chromosome number 2n = 36 was found for 14 species. Four sister species *N.albimarginatus*, *N.cardinalis*, *N.rubripinnis* and *N.ruudwildekampi* had similar karyotypes with 34 uniarmed and only 2 biarmed chromosomes (NF = 38). Karyotypes of three species *N.eggersi*, *N.korthausae*, and *N.wattersi* possesed 32 uniarmed and 4 biarmed chromosomes (NF = 40). Females of *N.guentheri* had more chromosome than males 2n = 36/35 and multiple sex chromosome system X_1_X_1_X_2_X_2_/X_1_X_2_Y type was revealed.

Karyotypes of other species had uniformly decreasing numbers of uniarmed chromosomes from 30 to 14 and numbers of biarmed chromosomes increased correspondingly.

Two species *N.foerschi* and *N.jubbi* had the 2n = 34 with 22 uniarmed and 12 biarmed chromosomes (NF = 46).

Only one species *N.kilomberoensis* possessed the 2n = 32 with karyotype formulae 8m+6sm+18st/a and NF = 46.

In karyotypes of two species *N.annectens* (2n = 28, NF = 36) and *N.lourensi* (2n = 27/28, NF = 34) uniarmed chromosomes dominated over biarmed ones. *N.lourensi* possessed multiple sex chromosome system X_1_X_1_X_2_X2/X_1_X_2_Y type.

*N.flammicomantis* possessed the lowest diploid numbers in the subgenus 2n = 20. The karyotype of *N.flammicomantis* consisted mainly of biarmed chromosomes with one pair of uniarmed chromosomes (NF = 38).

**Table 1.** Diploid chromosome numbers (2n), fundamental numbers (NF) and karyotype structures of analysed species. [*sex chromosome system of X_1_X_1_X_2_X_2_/X_1_X_2_Y type]

Species	2n	NF	Karyotype structure	Number of specimens karyotyped	References
Subgenus **Cynobranchius Costa, 2018**					
*N.microlepis* (Vinciguerra, 1897)	24	26	2m+22st/a		Scheel 1990
*N.fasciatus* Wildekamp & Haas, 1992	34	46	12msm+22st/a		Scheel 1981
**Subgenus Plesiobranchius Costa, 2018**					
*N.virgatus* Chambers, 1984	32	32	32st/a	2♀/2♂	This study
**Subgenus Nothobranchius Peters, 1868**					
*N.furzeri* Jubb, 1971	38	60	14m+8sm+16st/a	4♀/5♂	This study, Scheel 1981,^1990^; Reichwald et al. 2009
*N.kadleci* Reichard, 2010	38	62	16m+8sm+14st/a	3♀/5♂	This study
*N.krysanovi* Shidlovskiy, Watters & Wildekamp, 2010	18	34	8m+8sm+2st/a	3♀/5♂	This study, Shidlovskiy et al. 2010; Safronova and Krysanov 2015
*N.kuhntae* (Ahl, 1926)	38	52	6m+8sm+24st/a	1♀/1♂	This study
*N.orthonotus* (Peters, 1844)	38	48	8m+2sm+28st/a	2♀/3♂	This study, Scheel 1990
*N.pienaari* Shidlovskiy, Watters & Wildekamp, 2010	34	42	6m+2sm+26st/a	4♀/4♂	This study, Shidlovskiy et al. 2010
*N.rachovii* Ahl, 1926	16	30	8m+6sm+2st/a	10♀/12♂	This study, Ewulonu et al. 1985; Krysanov 1992; Shidlovskiy et al. 2010; Safronova and Krysanov 2015
**Subgenus Paranothobranchius Seegers, 1985**					
*N.ocellatus* Seegers, 1985	30	40	2m+8sm+20st/a	2 larvae	This study
**Subgenus Zononothobranchius Radda, 1969**					
*N.boklundi* Valdesalici, 2010	36	46	6m+4sm+26st/a	2♀/3♂	This study
*N.brieni* Poll, 1938*	50♀ 49♂	50♀ 50♂	♀50st/a ♂1m+48st/a	4♀/5♂	This study, Krysanov et al. 2016
*N.capriviensis* Watters, Wildekamp & Shidlovskiy, 2015	36	58	4m+18sm+14st/a	1♀/2♂	This study
*N.chochamandai* Nagy, 2014	36	64	18m+10sm+8st/a	5♀/7♂	This study
*N.flagrans* Nagy, 2014	36	48	10m+2sm+24st/a	3♀/4♂	This study
*N.hassoni* Valdesalici & Wildekamp, 2004	36	52	8m+8sm+20st/a	3♀/5♂	This study
*N.ivanovae* Valdesalici, 2012	36	64	22m+6sm+8st/a	3♀/3♂	This study
*N.kafuensis* Wildekamp & Rosenstock, 1989	36	66	8m+22sm+6st/a	1♀/2♂	This study, Scheel 1981, 1990
*N.kardashevi* Valdesalici, 2012	36	52	6m+10sm+20st/a	2♀/3♂	This study, Valdesalici 2015
*N.malaissei* Wildekamp, 1978	48	62	4m+10sm+34st/a	3♀/3♂	This study
*N.milvertzi* Nagy, 2014	38	54	10m+6sm+22st/a	4♀/4♂	This study
*N.neumanni* (Hilgendorf, 1905)	36	70	18m+16sm+2st/a	4♀/5♂	This study
*N.nubaensis* Valdesalici, Bellemans, Kardashev & Golubtsov, 2009	36	62	14m+12sm+10st/a	3♀/4♂	This study, Valdesalici 2015
*N.polli* Wildekamp, 1978	36	60	10m+14sm+12st/a	2♀/3♂	This study
*N.robustus* Ahl, 1935	36	58	4m+18sm+14st/a	1♂	This study, Wildekamp 2004
*N.rosenstocki* Valdesalici & Wildekamp, 2005	36	62	14m+12sm+10st/a	1♀/2♂	This study
*N.rubroreticulatus* Blache & Miton, 1960	36	58	12m+10sm+14st/a	2♀/2♂	This study
*N.seegersi* Valdesalici & Kardashev, 2011	36	56	8m+12sm+16st/a	4♀/4♂	This study
*N.steinforti* Wildekamp, 1977	36	56	10m+10sm+16st/a	2♀/3♂	This study, Scheel 1981, 1990
*N.streltsovi* Valdesalici, 2016	36	48	6m+6sm+24st/a	3♀/3♂	This study
*N.symoensi* Wildekamp, 1978	36	68	20m+12sm+4st/a	2♀/3♂	This study
*N.taeniopygus* Hilgendorf, 1891	36	66	14m+16sm+6st/a	4♀/5♂	This study
*N.ugandensis* Wildekamp, 1994	36	58	8m+14sm+14st/a	3♀/3♂	This study, Wildekamp 1994, Valdesalici 2015
**Subgenus Adiniops Myers, 1924**					
*N.albimarginatus* Watters, Wildekamp & Cooper, 1998	36	38	2m+34st/a	3♀/5♂	This study
*N.annectens* Watters, Wildekamp & Cooper, 1998	28	36	8m+20st/a	5♀/7♂	This study
*N.cardinalis* Watters, Cooper & Wildekamp, 2008	36	38	2m+34st/a	8♀/12♂	This study
*N.eggersi* Seegers, 1982	36	40	4m+32st/a	5♀/6♂	This study, Scheel 1990
*N.elongatus* Wildekamp, 1982	38	48	8m+2sm+28st/a	1♀/2♂	This study, Wildekamp 1982, Scheel 1990
*N.flammicomantis* Wildekamp, Watters & Sainthouse, 1998	20	38	18m+2st/a	5♀/8♂	This study
*N.foerschi* Wildekamp & Berkenkamp, 1979	34	46	10m+2sm+22st/a	3♀/5♂	This study, Scheel 1981, 1990; Ewulonu et al. 1985
*N.fuscotaeniatus* Seegers, 1997	38	40	2sm+36st/a	3♀/6♂	This study
*N.geminus* Wildekamp, Watters & Sainthouse, 2002	38	40	2sm+36st/a	2♀/3♂	This study
*N.guentheri* (Pfeffer, 1893) *	36♀ 35♂	40♀ 39♂	♀2m+2sm+32st/a ♂2m+2sm+31st/a	5♀/7♂	This study, Scheel 1990, Ewulonu et al. 1985
*N.hengstleri* Valdesalici, 2007	38	42	2m+2sm+34st/a	3♀/5♂	This study, Wildekamp et al. 2009
*N.interruptus* Wildekamp & Berkenkamp, 1979	36	50	8m+6sm+22st/a	2♀/3♂	This study
*N.janpapi* Wildekamp, 1977*	38♀ 37♂	48♀ 49♂	♀2m+8sm+28st/a ♂3m+9sm+25st/a	5♀/7♂	This study, Scheel 1990
*N.jubbi* Wildekamp & Berkenkamp, 1979	34	46	4m+8sm+22st/a	2♀/3♂	This study, Scheel 1981, 1990; Wildekamp 1982, Wildekamp et al. 1986
*N.kilomberoensis* Wildekamp, Watters & Sainthouse, 2002	32	46	8m+6sm+18st/a	2♀/4♂	This study
*N.kirki* Jubb, 1969	36	50	2m+12sm+22st/a	1♀/2♂	This study, Scheel 1981, 1990
*N.korthausae* Meinken, 1973	36	40	4m+32st/a	3♀/5♂	This study, Scheel 1981, 1990
*N.lourensi* Wildekamp, 1977*	28♀ 27♂	34♀ 34♂	♀6m+22st/a ♂7m+20st/a	2♀/3♂	This study
*N.lucius* Shidlovskiy, Watters & Wildekamp, 2010	36	58	6m+16sm+14st/a	2♀/3♂	This study, Wildekamp et al. 2009
*N.luekei* Seegers, 1984	38	40	2m+36st/a	2♀/2♂	This study
*N.makondorum* Shidlovskiy, Watters & Wildekamp, 2010	36	50	6m+8sm+22st/a	3♀/4♂	This study, Wildekamp et al. 2009
*N.melanospilus* (Pfeffer, 1896)	38	50	4m+8sm+26st/a	3♀/4♂	This study, Scheel 1981, 1990; Wildekamp et al. 2009
*N.palmqvisti* (Lönnberg, 1907)	36	42	6m+30st/a	2♀/2♂	This study, Ewulonu et al. 1985
*N.patrizii* (Vinciguerra, 1897)	36	52	4m+12sm+20st/a	2♀/2♂	This study, Ewulonu et al. 1985
*N.rubripinnis* Seegers, 1986	36	38	2m+34st/a	2♀/2♂	This study
*N.ruudwildekampi* Costa, 2009	36	38	2m+34st/a	3♀/4♂	This study
*N.vosseleri* Ahl, 1924	38	60	6m+16sm+16st/a	2♀/3♂	This study
*N.wattersi* Ng'oma, Valdesalici, Reichwald & Cellerino, 2013	36	40	4m+32st/a	2♀/2♂	This study, Scheel 1990
**Unrecognized species**					
N.ditte Nagy, 2018*	40♀ 39♂	♀64 ♂64	♀12m+12sm+16st/a ♂13m+12sm+14st/a	3♀/4♂	This study
N. torgashevi Valdesalici, 2015	36	46	6m+4sm+26st/a	3♀/4♂	This study, Valdesalici 2015
N. usanguensis Wildekamp, Watters & Shidlovskiy, 2014	36	54	6m+12sm+18st/a	1♀/2♂	This study
**Genus *Fundulosoma* Ahl, 1924**					
*Fundulosomathierryi* (Ahl, 1924) *	44♀ 43♂	46♀ 45♂	♀2m+42st/a ♂1m+1sm+41st/a	2♀/4♂	This study
**Genus *Pronothobranchius* Radda, 1969**					
*Pronothobranchiuskiyawensis* Ahl, 1928	28	30	2m+26st/a	2♂	This study

## Discussion

### Karyotype characteristics of representatives of the genus *Nothobranchius*

Karyotypes of 65 species of the genus *Nothobranchius* were overviewed and those of 35 species reported here for first time.

The results of present work have shown that representatives of the genus *Nothobranchius* possess a highly diverse karyotype. The 2n ranged from 16 to 50. The most frequent was 2n = 36 (35 species) and the second was 2n = 38 (13 species). similar karyotype diversity was found only for one closely related genus *Aphyosemion* Myers, 1924 among the family Cyprinodontiformes (Völker et al. 2008).

It has been shown that karyotypes of teleost fish consisted mainly of uniarmed or biarmed chromosomes (Molina et al. 2014). We did not find a similar trend in karyotype structure within the genus *Nothobranchius*. Fully acrocentric or metacentric karyotypes occurred as frequently as intermediate type. Such a high diversity of 2n and karyotype structure could be the result of many inter- and intrachromosomal rearrangements.

Scheel (1990) assumed that the karyotype evolution of the Old World Cyprinodontidae proceeded by decreasing the 2n while increasing the proportion of biarmed chromosomes in the karyotype by means of centric fusions. The correlation between the proportion of biarmed chromosomes and 2n was non-significant for the representatives of the genus *Nothobranchius* in contrast to those of the genus *Aphyosemion* (Agnèse et al. 2006) since pericentric inversions played essential role in the chromosome evolution of the genus.

### Sex chromosomes

Most of the studied species did not display morphologically distinguished sex chromosomes. Sex chromosomes were found only in six species, namely *N.guentheri* (Ewulonu et al. 1985), *N.brieni* (Krysanov et al. 2016), *N.lourensi*, *N.janpapi*, *N.ditte* and *F.thierryi* (this study) where multiple sex chromosome system of X_1_X_1_X_2_X_2_/X_1_X_2_Y type was found. Neo-Y chromosome likely originated through Robertsonian fusion of the original Y chromosome and autosome as was shown for another fish species (Kitano and Peichel 2012). Nothobranchius species with multiple sex chromosomes were found in two subgenera *Zononothobranchius* (*N.brieni*) and *Adiniops* (*N.guentheri*, *N.lourensi* and *N.janpapi*) (Costa 2018). According to molecular data *N.guentheri* and *N.janpapi* are not closely related (Dorn et al. 2014). We suppose that multiple sex chromosomes originated in these species independently.

### Chromosome evolution of *Nothobranchius* subgenera

Subgenera *Cynobranchius* and *Plesiobranchius* form basal Northern phylogenetic clade (sensu Dorn et al., 2014). It is noteworthy that the species with the most distinctive 2n and karyotype structures, namely *N.virgatus* and *N.microlepis* belonged to the basal clade.

Subgenus Nothobranchius corresponds well with the Southern clade (sensu Dorn et al 2014). Karyotype alterations by pericentric inversions were main trends in the karyotype evolution of species with 2n = 38. Four species *N.furzeri*, *N.kadleci*, *N.orthonotus* and *N.kuhntae* distinguished from each other by the ratio of uniarmed and biarmed chromosomes.

Reductions of diploid chromosomes number by fusions were probably characteristic of species with 2n lower 38. Biarmed chromosomes dominated in the karyotypes of species (*N.rachovii* and *N.krysanovi*) with the lowest diploid numbers (16 and 18) in the genus.

Only the species *N.ocellatus* from the subgenus Paranothobranchius with a distinctive karyotype structure is included in the Southern clade.

Subgenus Zononothobranchius corresponds well with the Inland clade (sensu Dorn et al 2014). Karyotypes of all species except *N.malaissei*, *N.brieni* and *N.milvertzi* have 2n = 36 and ratio of biarmed and uniarmed chromosomes differs among species. The karyotype evolution of the species with the 2n = 36 probably proceeded mainly by pericentric inversions.

Two species *N.malaissei* (2n=48), *N.brieni* (2n=49/50) had the highest diploid chromosome numbers among all species of the genus and high percent of uniarmed chromosomes.

Therefore, karyotype evolution of the subgenus proceeded mainly by pericentric inversions or rarest chromosome fusions (or fissions).

Subgenus Adiniops corresponds well with the Coastal clade (sensu Dorn et al 2014). Most species of the subgenus have diploid chromosomes number 36 or 38. And four species have diploid number lower than 36. Karyotype diversity is a result of chromosome fusions, fissions and pericentric inversions. Moreover, three species *N.guentheri*, *N.janpapi* and *N.lourensi* have multiple sex chromosome system.

Thus, two main trends were revealed in chromosome evolution of the genus: chromosome fusions (or rare fissions) and pericentric inversions.

## Conclusions

According to our data species of the genus *Nothobranchius* possess high variability of karyotype structure and diploid chromosome numbers. Such variability exists as a result of chromosome fusions or fissions and pericentric inversion, which is especially characteristic for the species with 2n equal 36 and 38. Centromere fusion apparently took place in formation of karyotypes with reduced 2n (less than 36).

In our opinion, variability of *Nothobranchius* karyotypes is associated with the characteristics of its life cycle and inhabiting in ephemeral partly isolated pools of East African savannah. Karyotype flexibility of *Nothobranchius* individuals may play adaptive role for survival under unstable conditions.
